# Supplementary material for: Berberine-Based Carbon Quantum Dots Improve Intestinal Barrier Injury and Alleviate Oxidative Stress in C57BL/6 Mice with 5-Fluorouracil-Induced Intestinal Mucositis by Enhancing Gut-Derived Short-Chain Fatty Acids Contents
Source: Molecules. 2023 Feb 24;28(5):2148. doi: 10.3390/molecules28052148 (PMC10004514; doi:10.3390/molecules28052148)
Supplement: Supplementary file 1 [file molecules-28-02148-s001.zip › molecules-2214120-supplementary.pdf]

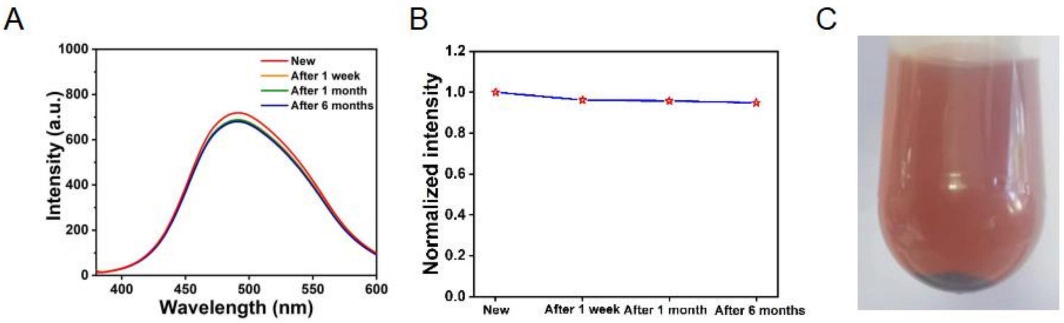

Figure S1. The performance and morphology of synthesized Ber-CDs. A: Fluorescence intensity of different wavelength of Ber-CDs with different preservation time (Samples synthesized for 0 week, 1 week, 1 month or 6 month). B: Maximum fluorescence intensity of Ber-CDs with different storage time (Samples synthesized for 0 week, 1 week, 1 month or 6 month). C: Representative morphology of synthesized Ber-CDs.
